# Acceptance of family doctors among residents in China: a cross-sectional study

**DOI:** 10.3389/fmed.2024.1435940

**Published:** 2024-09-05

**Authors:** Jing Feng, Zihui Lei, Xinyan Li, Ge Qu, Yuchao Sun, Yanling Zheng, Yanli Zuo, Yong Gan, Jun Ye

**Affiliations:** ^1^Department of Social Medicine and Health Management, School of Public Health, Tongji Medical College, Huazhong University of Science and Technology, Wuhan, China; ^2^Department of General Practice, Shouyilu Street Community Health Service Center, Wuhan, China; ^3^School of General Practice, Guangxi Medical University, Nanning, China; ^4^Department of Public Management, College of Medical Humanities and Management, Wenzhou Medical University, Wenzhou, China

**Keywords:** family doctors, residents, physician-patient relations, family doctor contract services, China

## Abstract

**Objectives:**

This study aimed to investigate the level of acceptance of family doctors (FDs) exhibited by residents in China.

**Methods:**

A cross-sectional study based on a structured self-administered questionnaire was conducted to investigate residents in eastern, central, and western China between September and December 2021. A multivariable stepwise logistic regression model was employed to identify the factors associated with health-seeking behavior after the signing of agreements concerning family doctor contract services (FDCS) as well as residents’ willingness to change FDs.

**Results:**

Among the 2,394 respondents included in this research, 55.8% sought primary care from their FDs when they became ill, whereas 9.7% expressed a willingness to change FDs. Residents who reported high levels of satisfaction with FDCS [odds ratio (OR) = 2.162] and trust in FDs (OR = 1.430) were more likely to seek initial help from FDs. In addition, residents from central China (OR = 0.546) and western China (OR = 0.704) and those who exhibited a high level of trust in FDs (OR = 0.238) were less likely to change FDs.

**Conclusion:**

The level of FD acceptance among Chinese residents was relatively high. Satisfaction with FDCS and trust in FDs were associated with the acceptance of FDs among residents. FDs should make efforts to enhance the quality of health services as well as the overall health experience of residents.

## Introduction

Primary healthcare (PHC) constitutes a whole-of-society approach to the task of providing optimal healthcare and services to everyone in all places. The Declaration of Alma-Ata, which was issued by the World Health Organization (WHO) in 1978, was the first to call for urgent and effective national and international action to be taken to develop and implement PHC worldwide (1). Forty years later, the Global Conference on Primary Health Care in Astana reaffirmed the role of PHC as the most effective and efficient approach to the tasks of achieving universal health coverage and meeting the Sustainable Development Goals (2). PHC, which is viewed as one of the most effective approaches to the task of ensuring health for all people, plays essential roles in disease prevention, treatment, and health promotion. According to the WHO, scaling up PHC interventions in low- and middle-income countries could save 60 million lives and increase average life expectancy by 3.7 years by 2030 (3).

The family doctor (FD) system, which is a crucial component of PHC, has been implemented in more than 50 countries. The United Kingdom, France, the Netherlands, and Indonesia have a “gatekeeper” system in which residents must designate a general practitioner as their FDs, and they must obtain a diagnosis from their FDs before receiving a referral (4–7). In contrast, there was no mandatory contracting requirement in the United States, Belgium, and Japan (8), where residents can voluntarily register with FDs and receive health care services (9, 10). Different countries have different models of FD services. However, the implementation of this system in China started relatively late and has progressed more slowly than in Western countries (11). China has implemented several favorable health policies aimed at enhancing the FD system. In 2009, China implemented a new phase of healthcare reform based on the pledge to develop a robust and efficient PHC system (12). The notion of family doctor contract services (FDCS) was initially introduced as a fundamental health policy aimed at enhancing PHC. In 2011, the State Council issued the Guiding Opinions on Establishing a General Practitioner System, which called for the establishment of service teams including general practitioners as core members, who would sign contracts and foster stable relationships with residents (13). In 2016, when the Guiding Opinions on Promoting Family Doctor Contract Services was released, FDCS was officially implemented nationwide (14). Since that time, numerous cities have sought to explore the effective implementation of FDCS. By 2020, the rate of FDCS contracts among target groups (including older adult individuals, pregnant women, children, disabled individuals, and people with chronic diseases) had increased from 28.33% in 2015 to 75.46%, and the percentage of county-level visits reached 94% in 2020, which represented an increase of 10% over 2015 (15). FDCS plays a crucial role in facilitating the transformation of the health service model from a treatment-centered approach to a health-centered approach.

FDCS represents the extension and advancement of community health services based on the principles of full notification, voluntary signing, and standardized service (16). Residents have the autonomy to choose and sign contracts with FDs voluntarily, while FDs provide contracted residents with basic medical services and public health services. Typically, these contracts are signed for a duration of one year, and upon its expiration, residents have the flexibility to renew their FDCS agreements with their current FDs, select a different FD, or conclude the contract (14). The decisions made by residents may reflect the quality of service provided by FDs and the effectiveness of FDCS to some extent (17).

**Figure 1:**
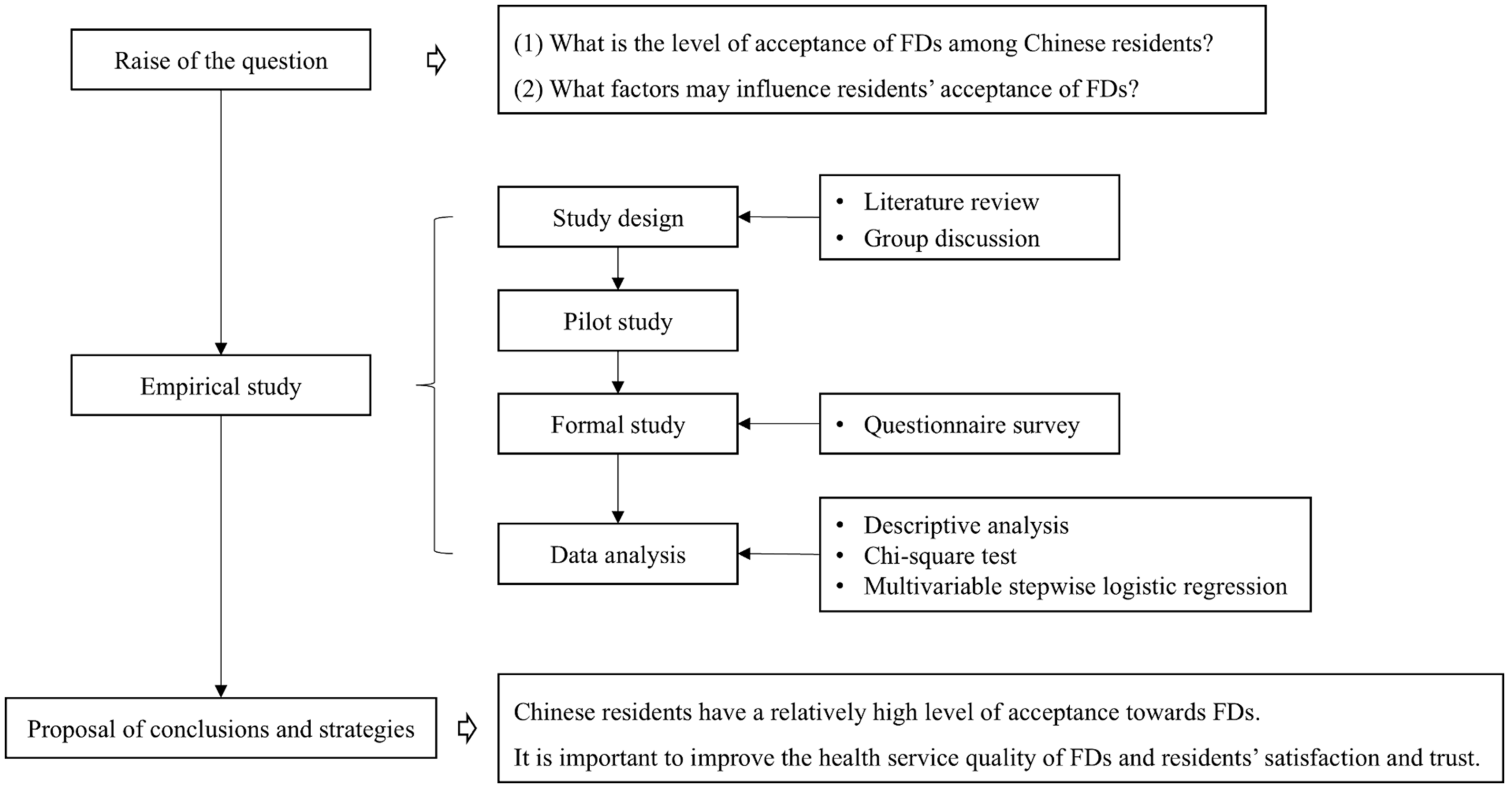
Flow chart for this study; FDs, Family doctors.

In the context of the high-quality development of FDCS, the signing rate has raised widespread attention. Numerous studies have examined residents’ contract behavior and their inclination to maintain their current contracts with FDs (16, 18–20), focusing on the impact of individual characteristics and satisfaction (21, 22). Most studies only investigated a particular province or city, limiting the generalizability of their findings. In the meta-analysis, the signing rate of residents with FDs varied across different regions (23), and satisfaction with FDs and their medical skills, and trust in FDs were positively associated with residents’ willingness to renew contracts (24). While increasing the signing and renewal rates was highly significant, the phenomenon of residents signing contracts but not making appointments was common due to their autonomy in seeking medical care (25). The implementation and continuity of FDCS are challenging to guarantee, and residents’ medical perceptions and behaviors after signing contracts remain underexplored. It is still unanswered whether FDs have achieved significant levels of acceptance among residents. This study aimed to fill this research gap. The following questions were proposed: (1) What is the level of acceptance of FDs among Chinese residents who signed contracts with FDs? (2) What factors may influence residents’ acceptance of FDs? A cross-sectional study was conducted in China with the goal of investigating residents’ health-seeking behaviors after signing agreements concerning FDCS, their willingness to change FDs, and the factors associated with these decisions. The flow chart of this study was shown in Figure 1. The findings of this research could provide evidence to improve cognition of FDCS among residents, enhance the service quality of FDs, and support the further development of FDCS in the context of PHC in China.

## Methods

### Study population and sampling

A cross-sectional study was conducted in China between September and December 2021. A multistage stratified sampling strategy was employed to recruit participants for this research. First, two cities each were selected from eastern China (Suzhou, Wenzhou), central China (Wuhan, Changsha), and western China (Nanning, Chongqing). Second, five to ten communities or villages (towns) were randomly selected from each chosen city. Third, 100 to 120 residents were randomly recruited from each community or village (town) to participate in an anonymous, self-administered questionnaire survey that was administered via WeChat. This study included residents aged ≥18 years who had resided in the area for at least 6 months; individuals with any reading problems or mental disorders were excluded.

**Figure 2:**
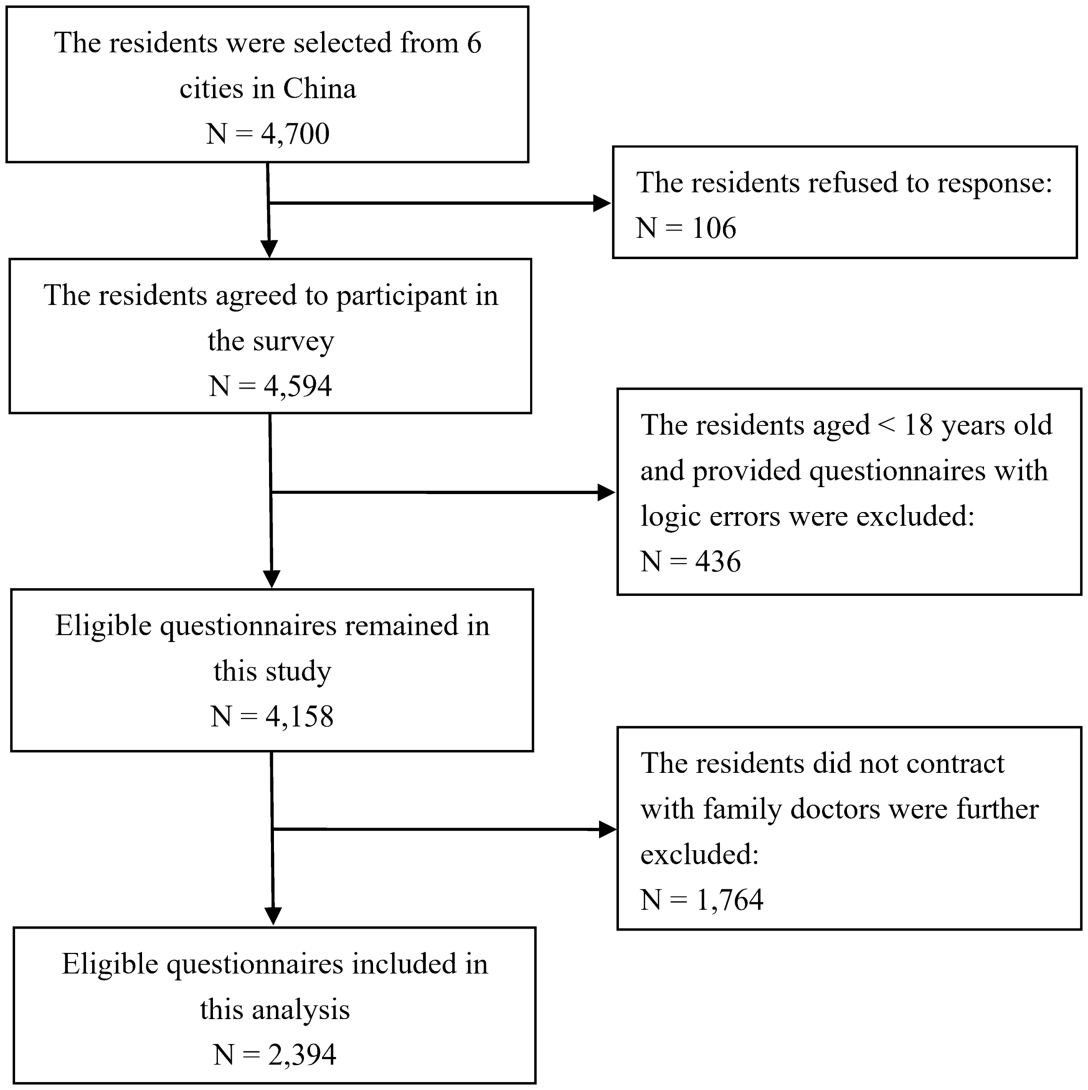
Flow chart for the sampling method used in this study.

A total of 4,700 residents were invited to participate in this survey. Among this group, 106 residents declined to respond. Therefore, 4,594 questionnaires were collected, for a response rate of 97.7%. A total of 436 residents who were aged <18 years or who provided questionnaires featuring logical errors were excluded. Only 2,394 participants were thus included in this analysis, which aimed to evaluate the acceptance of FDs from the perspective of residents who had signed contracts with FDs (Figure 2).

The study protocol was approved by the Ethics Committee of the Wenzhou Medical University Institutional Review Board, Wenzhou, China (no. 2021-019). Informed consent was obtained from all participants.

## Measurement

### Dependent variable

The acceptance of FDs was measured via one positive item and one negative item. (1) “How has your health-seeking behavior changed after signing an agreement concerning FDCS?” The responses included four options: seeking help from FDs first when one becomes sick and being more likely to visit primary health institutions (PHIs) than was previously the case; seeking help from FDs for minor illnesses and visiting the hospital for major illnesses; seeking help from FDs for healthcare and visiting the hospital when sick; and visiting the hospital as the first choice when one feels unwell. The first option was assigned a value of 1, while the remaining options were assigned a value of 0. (2) “Are you willing to change FDs within the next year?” The response options were categorized as follows: “yes” (which was assigned a value of 1), “no” (which was assigned a value of 0), and “not sure” (which was assigned a value of 0). Moreover, the reasons underlying residents’ willingness to change FDs were investigated and assessed via a closed-ended, multiple-choice question (i.e., more than one answer was allowed) that featured five response options (low level of health services, poor service attitude, imbalance between health service delivery and the resident’s demands, negative effects of the health service, and other).

### Independent variables

Four types of independent variables were included: (1) sociodemographic characteristics, including region, age, sex, ethnicity, marital status, residence, household registration, level of education, employment status, individual monthly income, number of family members living together, and medical insurance; (2) health-related factors, such as self-perceived health, smoking and alcohol drinking status, chronic disease, and illness during the past 2 weeks; (3) utilization and awareness of health services, such as individual annual medical expenditure, number of visits to PHIs during the past year, number of visits to hospitals during the past year, time required to walk to the nearest PHI, and awareness of primary health services; and (4) awareness and attitudes toward FDCS, including awareness of FDCS, satisfaction with FDCS, and trust in FDs.

FDCS satisfaction was assessed via a 5-point Likert scale ranging from 1 (very dissatisfied) to 5 (very satisfied) (26). This measure consisted of 10 items pertaining to 10 dimensions of satisfaction with FDCS. Total FDCS satisfaction scores ranged from 10 to 50, in which context higher scores indicated greater satisfaction. In this study, an overall score of 40 or above was considered to be an indicator of a high level of satisfaction with FDCS. The *Cronbach’s α* coefficient for this scale was 0.98.

The Chinese version of the Wake Forest Physician Trust Scale (WFPTS), which was originally developed by Halls et al. (27) and modified by Dong et al. (28), was utilized to assess residents’ trust in FDs (29). This scale contained 10 items that were scored on a 5-point Likert scale. Total scores of the scale ranged from 10 to 50, in which context higher scores indicated higher levels of trust in FDs. Respondents who scored 40 or higher were considered to exhibit high levels of trust in FDs. In this study, the *Cronbach’s α* coefficient for this scale was 0.75.

### Data collection and quality control

Quality control measures were implemented throughout the entire investigation process to ensure the authenticity and validity of the data. The questionnaire was designed on the basis of a literature review and group discussions. The questionnaire was pilot tested among 60 residents from a community and a village in Wuhan, Hubei Province. Necessary modifications were made on the basis of the valuable feedback provided by these residents. The electronic questionnaire was distributed to the residents who participated in this research via WeChat with the assistance of local PHIs and neighborhood committees. The data were automatically stored in a web-based database and subsequently downloaded and examined by the investigators.

### Statistical analysis

Statistical Package for Social Sciences (SPSS, Inc., Chicago, IL, Version 27.0) software was used to conduct all the data analyses. A descriptive analysis that involved calculating frequencies and percentages was conducted to describe the sample characteristics. A comparison of potential factors with health-seeking behavior after the signing of agreements concerning FDCS or willingness to change FDs was conducted by performing chi-square tests. A multivariable stepwise logistic regression model (the levels for selection and elimination were *p* = 0.05 and *p* = 0.10, respectively) was employed to explore the determinants of health-seeking behavior after the signing of agreements concerning FDCS or willingness to change FDs. Multicollinearity was assessed via the variance inflation factor (VIF). Typically, a VIF of 10 or larger suggests the presence of severe or serious collinearity (30). In this study, the VIF values ranged from 1.021 to 2.162, thus indicating that no collinearity was detected. All significance tests were two-tailed, and a value of *p* < 0.05 was considered to indicate statistical significance.

## Results

Within the sample, 24.3, 42.3, and 33.4% of the respondents were recruited from eastern, central, and western China, respectively. The age of the residents included in this research ranged from 18 to 97 years, and 42.8% of the participants were between the ages of 18 and 44 years. Females accounted for less than two-thirds (62.4%) of the sample. The majority of the respondents were Han Chinese people, married, living in urban areas, local, and employed. Only 26.9% of the residents had obtained a bachelor’s degree and above, and only 15.4% of the residents had an individual monthly income of more than 6,000 ¥. Slightly more than two-thirds of the residents lived with three or more family members and reported a good health status. Nearly all the residents (99.5%) possessed medical insurance. Fewer than one-fifth of the residents were smokers or drinkers at the time the survey was conducted. The proportions of participants who reported suffering from a chronic disease and experiencing illness in the past 2 weeks were 38.5 and 13.4%, respectively. Half of the respondents reported an individual annual medical expenditure of less than 1,000 ¥. Most of the respondents had visited PHIs or hospitals fewer than 3 times during the previous year. More than one-third of the respondents (36.8%) required more than 15 minutes to walk to the nearest PHI. The majority of the residents exhibited good awareness of primary health services and FDCS. The percentage of residents who reported a high level of satisfaction with FDCS was 82.5%, whereas 28.0% reported a high level of trust in FDs (Table 1).

**Table 1:** Descriptive statistics and univariate analysis of the differences among residents in terms of their health-seeking behavior after the signing of agreements concerning FDCS and their willingness to change FDs.

Variables	Total (%)	Seeking help from FDs initially when becoming sick (%)	*χ^2^*	Willingness to change FDs (%)	*χ^2^*
*Total*	2,394 (100.0)	1,337 (55.8)		233 (9.7)	
*Region*			11.848^*^		15.931^*^
Eastern China	581 (24.3)	360 (62.0)		77 (13.3)	
Central China	1,013 (42.3)	541 (53.4)		73 (7.2)	
Western China	800 (33.4)	436 (54.5)		83 (10.4)	
*Age in years*			7.184^*^		4.536
18–44	1,024 (42.8)	541 (52.8)		111 (10.8)	
45–64	835 (34.9)	492 (58.9)		82 (9.8)	
≥65	535 (22.3)	304 (56.8)		40 (7.5)	
*Sex*			11.438^*^		0.147
Male	901 (37.6)	543 (60.3)		85 (9.4)	
Female	1,493 (62.4)	794 (53.2)		148 (9.9)	
*Ethnicity*			10.740^*^		0.445
Han	2,253 (94.1)	1,277 (56.7)		217 (9.6)	
Other	141 (5.9)	60 (42.6)		16 (11.3)	
*Marital status*			2.303		0.128
Unmarried/divorced/widowed	316 (13.2)	164 (51.9)		29 (9.2)	
Married	2078 (86.8)	1,173 (56.4)		204 (9.8)	
*Residence*			4.044^*^		1.001
Urban	1,689 (70.6)	921 (54.5)		171 (10.1)	
Rural/suburban	705 (29.4)	416 (59.0)		62 (8.8)	
*Household registration*			2.866		0.370
Local	2,105 (87.9)	1,189 (56.5)		202 (9.6)	
Nonlocal	289 (12.1)	148 (51.2)		31 (10.7)	
*Level of education*			3.677		0.068
College degree or below	1750 (73.1)	998 (57.0)		172 (9.8)	
Bachelor’s degree or above	644 (26.9)	339 (52.6)		61 (9.5)	
*Employment status*			0.264		0.354
Employed	1745 (72.9)	969 (55.5)		166 (9.5)	
Unemployed/retired	649 (27.1)	368 (56.7)		67 (10.3)	
*Individual monthly income in ¥*			0.848		0.559
≤6,000	2025 (84.6)	1,139 (56.2)		201 (9.9)	
>6,000	369 (15.4)	198 (53.7)		32 (8.7)	
*Number of family members in residence*			3.277		3.789
1	138 (5.8)	81 (58.7)		14 (10.1)	
2	631 (26.4)	369 (58.5)		49 (7.8)	
≥3	1,625 (67.9)	887 (54.6)		170 (10.5)	
*Medical insurance*			2.057		0.898
Yes	2,383 (99.5)	1,328 (55.7)		231 (9.7)	
No	11 (0.5)	9 (81.8)		2 (18.2)	
*Self-perceived health*			4.319		4.603
Good	1,616 (67.5)	926 (57.3)		170 (10.5)	
Fair	597 (24.9)	314 (52.6)		52 (8.7)	
Bad	181 (7.6)	97 (53.6)		11 (6.1)	
*Smoking status*			5.528^*^		0.076
Former/never smoker	2009 (83.9)	1,101 (54.8)		197 (9.8)	
Current smoker	385 (16.1)	236 (61.3)		36 (9.4)	
*Alcohol consumption*			2.347		0.511
Former/never drinker	1984 (82.9)	1,094 (55.1)		197 (9.9)	
Current drinker	410 (17.1)	243 (59.3)		36 (8.8)	
*Chronic disease*			1.982		1.172
Yes	921 (38.5)	531 (57.7)		82 (8.9)	
No	1,473 (61.5)	806 (54.7)		151 (10.3)	
*Illness during the past 2 weeks*			0.156		2.465
Yes	321 (13.4)	176 (54.8)		39 (12.1)	
No	2073 (86.6)	1,161 (56.0)		194 (9.4)	
*Individual annual medical expenditure, ¥*			0.897		0.442
≤1,000	1,197 (50.0)	679 (56.7)		121 (10.1)	
1,001-5,000	930 (38.8)	514 (55.3)		86 (9.2)	
>5,000	267 (11.2)	144 (53.9)		26 (9.7)	
*Number of visits to PHIs during the past year*			18.818^*^		1.513
≤3	1,525 (63.7)	801 (52.5)		157 (10.3)	
>3	869 (36.3)	536 (61.7)		76 (8.7)	
*Number of visits to hospitals during the past year*			0.519		3.380
≤3	2030 (84.8)	1,140 (56.2)		188 (9.3)	
>3	364 (15.2)	197 (54.1)		45 (12.4)	
*Time required to walk to the nearest PHI in minutes*			20.899^*^		0.697
≤15	1,512 (63.2)	898 (59.4)		153 (10.1)	
>15	882 (36.8)	439 (49.8)		80 (9.1)	
*Awareness of primary health services*			49.736^*^		1.973
Good	1999 (83.5)	1,180 (59.0)		199 (10.0)	
Fair	350 (14.6)	139 (39.7)		28 (8.0)	
Bad	45 (1.9)	18 (40.0)		6 (13.3)	
*Awareness of FDCS*			79.482^*^		5.350
Good	1913 (79.9)	1,154 (60.3)		191 (10.0)	
Fair	394 (16.5)	156 (39.6)		29 (7.4)	
Bad	87 (3.6)	27 (31.0)		13 (14.9)	
*Satisfaction with FDCS*			91.948^*^		0.003
Low	418 (17.5)	145 (34.7)		41 (9.8)	
High	1976 (82.5)	1,192 (60.3)		192 (9.7)	
*Trust in FDs*			34.677^*^		46.268^*^
Low	1723 (72.0)	898 (52.1)		212 (12.3)	
High	671 (28.0)	439 (65.4)		21 (3.1)	

FDs, Family doctors; FDCS, Family doctor contract services; PHIs, Primary health institutions.

Among the 2,394 residents surveyed, more than half (55.8%) reported initially seeking help from their FDs when they became ill and expressed a greater willingness to visit PHIs after signing contracts with FDs, while 233 individuals (9.7%) expressed a willingness to change their FDs. The results of the chi-square tests revealed statistically significant differences in health-seeking behavior after the signing of an agreement concerning FDCS with regard to region, age, sex, ethnicity, residence, smoking status, number of visits to PHIs during the past year, time required to walk to the nearest PHI, awareness of primary health services, awareness of FDCS, satisfaction with FDCS, and trust in FDs (*p* < 0.05). Additionally, respondents’ willingness to change FDs exhibited significant differences with respect to region and trust in FDs (*p* < 0.05) (Table 1). Residents who had a high level of trust in FDs particularly preferred FDs as their first choice (65.4%) and were the least likely to change their FDs (3.1%). Table 2 presents the reasons for residents’ willingness to change FDs, revealing that the “low level of health services” was the main reason. “Unbalance between health services deliveries and demands in residents” was another important reason.

**Table 2:** Distribution of the reasons underlying residents’ willingness to change FDs (*N* = 233).

Items	*N*	%
Low level of health services	105	45.1
Poor service attitudes	64	27.5
Imbalance between health services deliveries and residents’ demands	91	39.1
Poor health service effects	60	25.8
Other	54	23.2

FDs, Family doctors.

All the variables contained in Table 1 were included in the multivariable stepwise logistic regression model, and the results are presented in Tables 3, 4. Residents who visited PHIs more than 3 times during the past year [odds ratio (OR) = 1.439] or who reported high levels of satisfaction with FDCS (OR = 2.162) and trust in FDs (OR = 1.430) were more likely to seek help from FDs initially when they became sick. Conversely, residents from central China (OR = 0.728), females (OR = 0.748), residents with non-Han ethnicities (OR = 0.672), those who visited hospitals more than 3 times during the past year (OR = 0.757), those who required more than 15 minutes to walk to the nearest PHI (OR = 0.735), and those who reported fair (OR = 0.511) or poor (OR = 0.408) awareness of FDCS were less likely to seek help from FDs initially when they became sick. Furthermore, residents from central China (OR = 0.546) or western China (OR = 0.704) as well as those who reported high levels of trust in FDs (OR = 0.238) were less likely to change FDs.

**Table 3:** Multivariable stepwise logistic regression models for associations with health-seeking behaviors after the signing of agreements concerning FDCS among residents.

Variables	*Β*	*SE*	*Wald χ^2^*	*P*	OR	95% CI
*Region (reference: eastern China)*						
Central China	−0.318	0.112	8.069	0.005	0.728	0.585, 0.906
Western China	−0.167	0.121	1.900	0.168	0.846	0.668, 1.073
*Sex (reference: male)*						
Female	−0.290	0.090	10.400	0.001	0.748	0.627, 0.892
*Ethnicity (reference: Han)*						
Other	−0.398	0.190	4.383	0.036	0.672	0.463, 0.975
*Number of visits to PHIs during the past year (reference: ≤3)*						
>3	0.364	0.098	13.763	<0.001	1.439	1.187, 1.744
*Number of visits to hospitals during the past year (reference: ≤3)*						
>3	−0.279	0.130	4.624	0.032	0.757	0.587, 0.976
*Time required to walk to the nearest PHI (reference: ≤15 minutes)*						
>15 minutes	−0.307	0.089	11.822	0.001	0.735	0.617, 0.876
*Awareness of FDCS (reference: good)*						
Fair	−0.671	0.119	31.736	<0.001	0.511	0.405, 0.645
Bad	−0.896	0.246	13.227	<0.001	0.408	0.252, 0.661
*Satisfaction with FDCS (reference: low)*						
High	0.771	0.120	40.975	<0.001	2.162	1.707, 2.738
*Trust in FDs (reference: low)*						
High	0.357	0.101	12.649	<0.001	1.430	1.174, 1.741
*Constant*	0.069	0.159	0.187	0.665	1.071	

CI, Confidence interval; FDs, Family doctors; FDCS, Family doctor contract services; OR, Odds ratio; PHIs, Primary health institutions.

**Table 4:** Multivariable stepwise logistic regression models for associations with residents’ willingness to change FDs.

Variables	*Β*	*SE*	*Wald χ^2^*	*P*	OR	95% CI
*Region (reference: eastern China)*						
Central China	−0.605	0.174	12.025	<0.001	0.546	0.388, 0.769
Western China	−0.350	0.170	4.227	0.040	0.704	0.504, 0.984
*Trust in FDs (reference: low)*						
High	−1.436	0.235	37.424	<0.001	0.238	0.150, 0.377
*Constant*	−1.624	0.126	165.493	<0.001	0.197	

CI, Confidence interval; FDs, Family doctors; OR, Odds ratio.

## Discussion

Previous research on perceptions and attitudes towards FDs focused on satisfaction. This study, for the first time, addressed the residents’ acceptance of FDs, revealing their healthcare-seeking behavior after signing contracts and willingness to change FDs. It was of great significance to evaluate the doctor-patient relationship and the effectiveness of FDCS. Additionally, different from previous studies limited to specific regions, the study population of this study covered the eastern, central, and western regions of China, providing more generalizable evidence.

In this study, more than half of the residents identified seeking help from FDs as their initial choice when they became sick after signing agreements concerning FDCS. Additionally, the majority of residents exhibited good awareness of FDCS and were unwilling to change FDs. These findings indicate that Chinese residents exhibit relatively high levels of acceptance of FDs; this situation is conducive to the development of FDCS. The first research question has been validated. Regarding the second research question, several factors were found to be associated with the acceptance of FDs, including region, awareness of awareness of FDCS, satisfaction with FDCS, and trust in FDs, etc.

The percentage of respondents who reported seeking initial help from their FDs was slightly lower than the corresponding figure among residents who signed service contracts with FDs in Shenzhen, China (61.9%) (31), while was much higher than the public in Nanjing, China (16.4%) (32). It has been proved that signing service contracts with FDs can significantly increase the probability of using PHIs as the first choice (31–33), and this positive effect of FDCS was also reflected in this study. Overall, nearly one-tenth of the participants in that study expressed a willingness to change their FDs. This proportion was higher than has been reported in findings collected in Slovenia, where only 2.0% of respondents reported considering changing their FDs in the near future (34). Differences in study location, sample size, sociocultural background, and healthcare system may explain these variations. However, the implementation of FDCS in China continues to offer room for improvement.

This investigation revealed that the primary reason for residents’ willingness to change FDs was the “low level of health services.” Similar results were found in previous studies. Distrust of FDs’ medical skills was the top reason accounting for the reluctance of residents to contract with FDs (21), and satisfaction with the medical skill of FDs was significantly associated with the renewal rate (24). The level of health service has been widely recognized to be associated with the ability to retain current customers and attract new customers as well as decreased costs and enhanced images on the part of medical institutions. Managers should thus offer more learning opportunities and training activities to improve the service level of FDs, thereby improving the overall quality of the services they provide. Additionally, nearly two-fifths of respondents chose the “unbalance between health services deliveries and demands in residents” as the reason for changing their FDs. This indicated that FDCS should be demand-oriented, deeply understand residents’ actual needs, and provide personalized services.

Satisfaction with FDCS was identified as the most significant factor associated with health-seeking behavior with respect to FDs, with an OR of 2.162. Previous studies have reported that patient satisfaction with received health services had a significantly positive effect on patient loyalty (35, 36). Patient loyalty referred to the inclination of individuals to choose the same healthcare providers consistently with regard to their future health service requirements due to their satisfaction with their past experience (37). Therefore, satisfaction with FDCS indicated a high level of acceptance of FDs to some extent. Furthermore, a lack of awareness regarding FDCS has been reported to be associated with a lower probability of health-seeking behavior from FDs, which was consistent with previous studies (22, 31). This finding highlights the necessity of publicizing and promoting FDCS. Diverse methods of health education and advertising such as lectures, television, and social media should be implemented to publicize the efficiency and advantages of FDCS.

Residents who reported higher levels of trust in their FDs were more likely to seek help from FDs initially and were less inclined to consider changing their FDs. Several studies have demonstrated the crucial role played by trust in the doctor–patient relationship (38–40). Trust serves as a subjective and direct indicator that reflects residents’ genuine sentiments toward their FDs; thus, residents who trust their FDs are more prone to maintain their contracts (19). It is essential to promote the implementation of the hierarchical medical system, improve the level of services provided by FDs, and gain the trust of residents.

Residents from central China and western China reported lower likelihoods of changing FDs than did respondents from eastern China. This disparity can be attributed to the significant inequality in healthcare resources across various regions of China (41). In 2022, the number of general practitioners per 10,000 population was 4.16 in eastern China, whereas the corresponding figures were 3.42 and 2.69 in central and western China, respectively (42). Due to the abundance of rich and high-quality healthcare resources in eastern China, residents in this region had greater access to healthcare services. Consequently, when residents were dissatisfied with the service provided by FDs, they were more inclined to seek alternative FDs who can offer services of a superior quality.

The proportion of respondents who reported initially seeking help from FDs when they became sick was higher among residents who were 65 years old or older (56.8%) than among respondents who were between the ages of 18 and 44 years (52.8%). Moreover, the results revealed a decrease in residents’ willingness to change FDs due to age; this value decreased from 10.8% among individuals who were between the ages of 18 and 44 years old to 7.5% among individuals who were 65 years or older. Older adult individuals are typically more susceptible to illnesses than are younger individuals, which may motivate the former group to seek targeted and continuous healthcare from their FDs (43). In addition, there is a special focus of FDCS policies on vulnerable groups, particularly older adult individuals, and the high signing rate among this group indicated the remarkable success of FDCS. In such cases, FDs seek to encourage older people to maintain their contracts (19).

Rural or suburban residents were more likely to seek initial help from FDs when they became sick than were urban residents, and the rate of willingness to change FDs exhibited by rural or suburban residents was similarly lower. The allocation of medical resources may affect residents’ utilization of health services. The abundant medical resources concentrated in urban and convenient medical environments made residents more willing to seek medical treatment in hospitals (22, 44). Additionally, interpersonal relationships in urban areas frequently involved interactions with strangers, whereas in rural areas, most interpersonal relationships were formed with acquaintances (45). With respect to the professional backgrounds of village doctors, most of these individuals were locals who were familiar with their patients’ economic status, family health history, and medical conditions (18). Consequently, these FDs effectively served as “gatekeepers of residents’ health” and established close relationships with residents, which may enhance residents’ utilization of FDCS and willingness to continue to sign contracts with their FDs.

### Strengths and limitations

The present study has several strengths. First, this study is the first to investigate health-seeking behavior among Chinese residents after the signing of agreements concerning FDCS as well as their willingness to change their FDs; it also explores the determinants of these factors. Second, the study offers novel insights into ways of evaluating the relationships between FDs and residents from the perspective of residents’ acceptance of FDs. Third, this study is a multi-city, large-sample research study that improves generalizability in a Chinese setting.

Nevertheless, this study has several limitations. First, the correlations observed among variables in this cross-sectional study do not allow causal conclusions to be drawn. Second, self-report bias may impact the results of this study, as all the data used in this research were self-reported.

### Implications for research and practice

This pioneering research, which was conducted in China, provides insights into Chinese residents’ acceptance of FDs based on a large-scale cross-sectional study that was conducted at the national level. According to the results of this study, serious attention should be given to the task of improving the quality of health services offered by FDs. Additionally, it is crucial to establish and strengthen trust in the context of relationships between FDs and residents.

In the future, a longitudinal study should be conducted to enable future researchers to draw causal inferences in this context. Furthermore, future studies should consider incorporating other potential factors, and the mechanism underlying the impacts of various influencing factors on residents’ acceptance of FDs should be explored.

## Conclusion

This study reveals that Chinese residents exhibit relatively high levels of acceptance of FDs. To promote the high-quality development of FDCS, it is imperative to enhance the health service delivery level of FDs and to foster satisfaction and trust among residents.

## Data availability statement

The raw data supporting the conclusions of this article will be made available by the authors, without undue reservation.

## Ethics statement

The studies involving humans were approved by the Ethics Committee of the Wenzhou Medical University Institutional Review Board. The studies were conducted in accordance with the local legislation and institutional requirements. The participants provided their oral informed consent to participate in this study.

## Author contributions

JF: Data curation, Investigation, Methodology, Writing – original draft, Writing – review & editing. ZL: Investigation, Writing – review & editing. XL: Investigation, Writing – review & editing. GQ: Investigation, Writing – review & editing. YS: Investigation, Writing – review & editing. YZh: Investigation, Writing – review & editing. YZu: Methodology, Writing – review & editing. YG: Conceptualization, Methodology, Supervision, Writing – review & editing, Project administration. JY: Conceptualization, Methodology, Supervision, Writing – review & editing, Funding acquisition, Project administration.
